# Bone-targeted mitochondrial delivery via magnetic-temperature responsive artificial cells for repairing age-related fractures

**DOI:** 10.3389/fphar.2025.1725973

**Published:** 2026-01-02

**Authors:** Shihao Nie, Yue Yu, Rong Yan, Taoran Liu, Yue Du, Zhuojing Luo, Shengyou Li, Jinghui Huang

**Affiliations:** 1 Department of Orthopaedics, Xijing Hospital, Fourth Military Medical University, Xi’an, China; 2 State Key Laboratory of Holistic Integrative Management of Gastrointestinal Cancers and National Clinical Research Center for Digestive Diseases, Xi’an, Shaanxi, China; 3 Institute of Medical Research, Northwestern Polytechnical University, Xi’an, China

**Keywords:** age-related fractures, artificial cells, anti-aging, mitochondria, magnetic-temperature responsive

## Abstract

**Background:**

Age-related bone diseases, such as osteoporosis and degenerative joint disorders, pose a significant global health challenge, leading to over 9 million fractures annually, which not only diminishes quality of life but also imposes a substantial socioeconomic burden on healthcare systems. A major clinical obstacle in the aging population is the significantly reduced regenerative capacity of bone, often resulting in delayed fracture healing or nonunion fractures. Mitochondria, as the central regulators of cellular energy metabolism, are essential for determining cell fate and supporting tissue regeneration. However, age-associated mitochondrial dysfunction critically impairs these processes. While transplanting healthy mitochondria is a promising therapeutic strategy, its efficacy is severely limited by poor targeting efficiency and inherent fragility of mitochondria in circulation. Developing an efficient mitochondrial transplantation for elderly fractures is of great importance.

**Methods:**

We constructed artificial cell microspheres (Fmito@ACs) containing mitochondria of fetal mouse mesenchymal stem cells and conducted systematic characterization of them. In vitro experiments evaluated the effects of Fmito@ACs on the functions of primary osteoblasts, and its role in delaying cellular senescence was analyzed through β-galactosidase staining and immunofluorescence analysis of senescence markers (P21 and γH2A.X). Its ability to restore mitochondrial function was assessed by measuring ROS, morphology, and energy metabolism. In animal experiments, labeled Fmito@ACs were tracked using IVIS Spectrum system, and their targeted accumulation at fracture sites guided by an external magnetic field was verified. The biosafety of the system was evaluated via H&E staining and hepatic/renal function parameters. Bone healing was monitored via micro-CT, X-ray, and histology on days 7, 14, and 21, while related gene expression and molecular mechanisms were analyzed by qPCR and transcriptome sequencing.

**Results:**

Fmito@ACs were successfully constructed and characterized, indicating a protective effect on mitochondria. The system ameliorated senescence in aged BMSCs, promoting osteogenesis by enhancing mitochondrial fusion and aerobic glycolysis. In an aged fracture model, Fmito@ACs showed targeted accumulation and biosafety, significantly improving healing.

**Conclusion:**

As an efficient mitochondrial-targeted delivery system, Fmito@ACs fully exploits the anti-aging effects of young mitochondria, providing a new strategy and theoretical basis for the treatment of age-related fractures.

## 1 Introduction

Aging is an irreversible biological process marked by progressive degenerative changes in the structural and functional reserves of organs and tissues (Childs et al., 2017). It leads to multiple age-related diseases, among which osteoporotic fractures are particularly prevalent due to their high incidence and severity (Wu et al., 2021). Bone marrow mesenchymal stem cells (BMSCs) play a key role in bone repair and regeneration owing to their multidirectional differentiation potential. However, aging significantly impairs the self-renewal, differentiation, and repair capacity of BMSCs, resulting in delayed fracture healing or nonunion after fractures, which severely affects the quality of life and life expectancy of elderly patients (Clark et al., 2020; Cheng et al., 2020).

**SCHEME 1:**
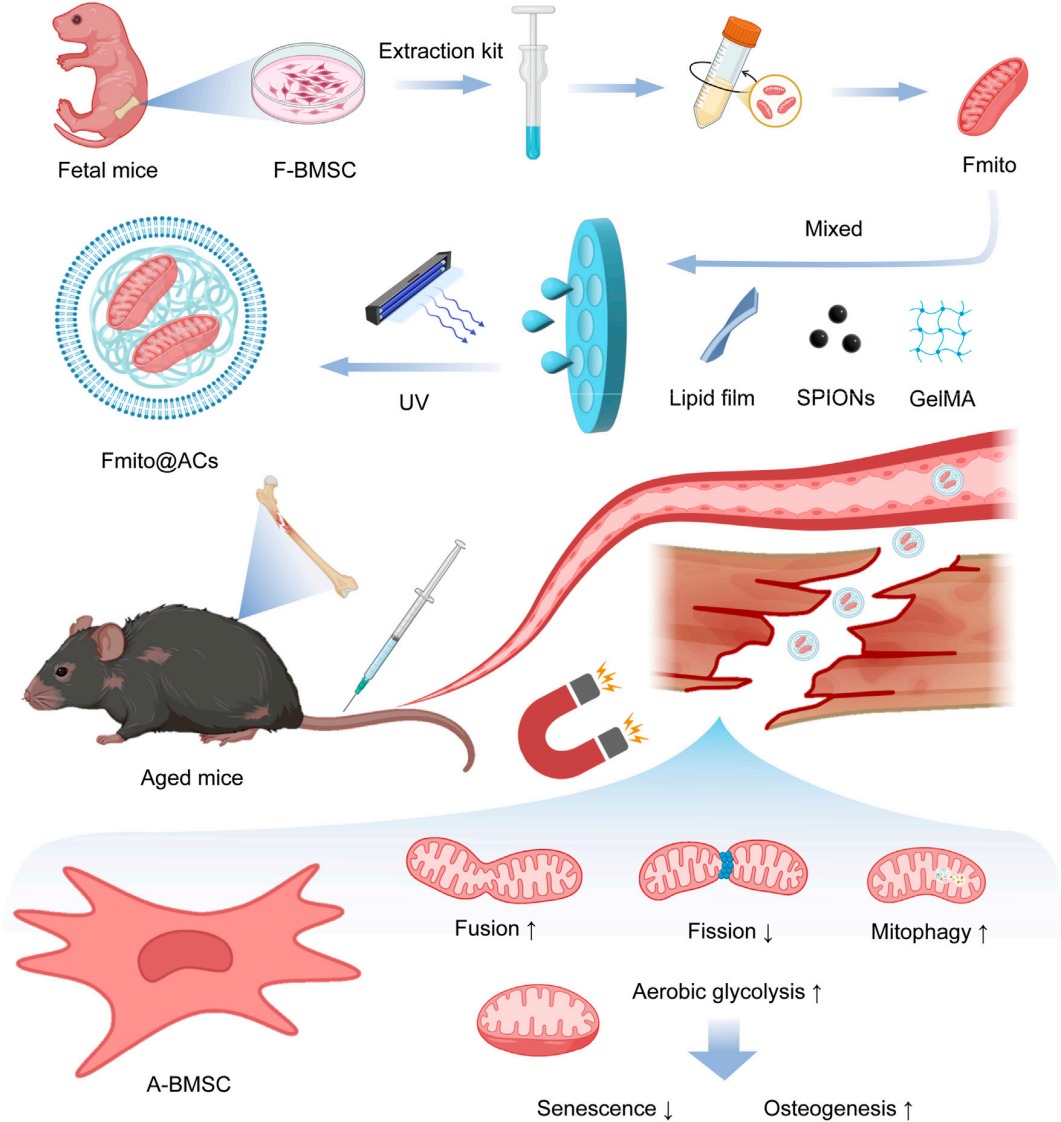
Schematic of the preparation of Fmito@ACs and their therapeutic effect in improving mitochondrial function of A-BMSCs to promote age-related fracture repair upon *in vivo* injection.

Bone regeneration is an energy-intensive process (Lecka-Czernik et al., 2025). Mitochondria, as the core regulators of cellular energy metabolism, generate ATP through oxidative phosphorylation to support osteogenesis and metabolic balance. BMSC dysfunction is closely linked to mitochondrial homeostasis imbalance (Guo et al., 2020). Aged bone marrow mesenchymal stem cells (A-BMSCs) exhibit evident mitochondrial abnormalities, including impaired mitophagy, disrupted energy metabolism, imbalance of fission and fusion, and formation of giant mitochondria. These changes activate senescence-associated pathways, exacerbate bone marrow inflammation, and impair regenerative potential (van Hameren et al., 2023; Gill et al., 2019; Ambrosi et al., 2021). In addition, the hypoxic microenvironment that develops after fracture worsens mitochondrial dysfunction and further weakens the regenerative ability of BMSCs (Liu et al., 2024). Therefore, targeted repair of mitochondrial function is essential to restore regeneration after aging-related fractures.

Exogenous supplementation with young mitochondria can partially restore the physiological function of senescent cells, offering potential therapeutic value (Nuerlan et al., 2025). However, conventional mitochondrial transfer methods face major limitations, including poor targeting, rapid clearance by the mononuclear phagocytic system, and susceptibility to oxidative damage in circulation, leading to insufficient accumulation at the injury site and limited efficacy (Wu et al., 2018). To address these issues, this study designed a novel targeted mitochondrial delivery system to achieve efficient and precise enrichment of functional mitochondria from fetal BMSCs (Fmito) at fracture sites, avoiding systemic clearance and metabolic attack while maximizing repair potential. This strategy provides a promising therapeutic approach for aging-related osteoporotic fractures with important implications for both basic research and clinical translation.

## 2 Methods

### 2.1 A-BMSCs isolation

C57BL/6 mice (age: 18 months) were euthanized via cervical dislocation and sterilized by immersion in 75% ice-cold ethanol for 5 min. The bilateral femurs and tibiae were aseptically dissected, and the surrounding muscle tissues were thoroughly removed. The epiphyses of the bones were cut off, and the bone marrow cavity was flushed with complete culture medium using a syringe until the bones turned white. The collected cell suspension was filtered through a 70-μm cell strainer into a tube and then centrifuged at 1,000 rpm for 5 min. The cells were resuspended in a complete medium and then seeded into culture dishes at a density of 1 × 10^7^ cells/dish. The cells were cultured in a humidified incubator at 37 °C with 5% CO_2_ atmosphere. The first complete medium change was performed after 24 h to remove the non-adherent cells. Subsequently, the medium was replaced every 2–3 days. When the cells reached 80%–90% confluence, they were passaged with 0.25% trypsin. Primary (P0) cells were typically ready for the first passage after approximately 7–10 days of culture.

### 2.2 A-BMSCs osteogenic differentiation

BMSCs were passaged 3–5 times under good growth conditions and seeded at an appropriate density. When the cells reached 70% confluence, the osteogenic induction was initiated. Alkaline phosphatase (ALP) staining and Alizarin Red S (ARS) staining were performed after 7 and 14 days of induction, respectively. The staining results were then statistically analyzed using ImageJ software.

### 2.3 Primary osteoclast culture and tartrate-resistant acid phosphatase (TRAP) staining

The femurs and tibiae were isolated from the mice, and the bone marrow cavity was flushed with α-MEM medium (Gibco, USA). The cell suspension was cultured for 24 h. After removing the medium, the remaining adherent cells were further cultured in α-MEM medium containing 50 ng/mL M-CSF (Amizona Scientific) for 3 days. Bone marrow-derived macrophages (BMDMs) were then carefully scraped with a cell scraper and seeded in a 96-well plate at a density of 1 × 10^5^ cells/well. Osteoclast differentiation was induced in an α-MEM medium containing 50 ng/mL M-CSF and 50 ng/mL RANKL (Amizona Scientific) for 5 days. A TRAP staining kit (Amizona Scientific) was used for staining. The cells containing multiple nuclei were identified as osteoclasts and counted microscopically.

### 2.4 Mitochondrial isolation

The mitochondria were freshly isolated from cells following the manufacturer’s protocol by using the Mitochondria Isolation Kit (Beyotime, C3601). Briefly, freshly harvested BMSCs from fetal mice were resuspended in pre-chilled mitochondria isolation reagent and incubated on an ice bath for 10–15 min. The cell suspension was homogenized with a glass homogenizer and then subjected to sequential centrifugation. The initial low-speed spin (600 × *g*, 10 min, 4 °C) was followed by a high-speed centrifugation (11,000 × *g*, 10 min, 4 °C) of the resulting supernatant. The final pellet, enriched with mitochondria, was resuspended in a mitochondrial storage buffer.

### 2.5 ACs preparation and characterization

Firstly, in order to form the biomimetic cell membrane shell, microspheres were prepared by the thin film hydration extrusion method. In brief, egg phosphatidylcholine (EggPC) and cholesterol (Chol) were dissolved in chloroform at an optimized molar ratio. The solution was rotary-evaporated and vacuum-dried for 12 h to form a uniform lipid membrane. The membrane was hydrated in phosphate buffer solution (PBS) at 37 °C for 30 min, and then centrifuged to collect the lipid membrane. Subsequently, in order to construct a photocrosslinkable hydrogel core and integrate the magnetic components into it, the isolated mitochondria Fmito were dispersed together with superparamagnetic iron oxide nanoparticles (SPIONs) and photocrosslinkable LAP in a 5% methacrylated gelatin (GelMA) solution. The mixture was extruded through a 1 µm polycarbonate membrane to obtain uniformly sized microcapsules. Finally, in order to permanently lock the structure of the microspheres and achieve their mechanical stability, the microcapsules were diluted with PBS appropriately, cross-linked for 180 s under a 405 nm ultraviolet light (UV), and then centrifuged and washed for collection to obtain the final microspheres. The zeta potential of the liposomes was detected by dynamic light scattering (DLS). The hydrodynamic particle size distribution of the microspheres in the aqueous solution was determined by a nanoparticle size analyzer (Nanosizer, PSS, Nicomp N3000). The magnetic hysteresis loop of ACs was measured using a vibrating sample magnetometer (LakeShore 7,404) to characterize their magnetic properties.

### 2.6 Western blot (WB) analysis

Total protein was extracted from A-BMSCs in a RIPA lysis buffer containing protease inhibitors. The proteins were separated by sodium dodecyl sulfate (SDS)-polyacrylamide gel electrophoresis (SDS-PAGE) and transferred onto a polyvinylidene fluoride (PVDF) membrane by using a wet transfer system. The membrane was blocked with 5% skim milk at room temperature for 2 h, followed by incubation with primary and secondary antibodies. The primary antibodies used were anti-OCN (20277-1-AP, Proteintech), anti-RUNX2 (82636-2-RR, Proteintech), anti-DRP1 (12957-1-AP, Proteintech), anti-FIS1 (10956-1-AP), anti-OPA1 (DF8587, Affinity), anti-MFN2 (DF8106, Affinity), anti-PINK1 (DF7742, Affinity) and anti-Parkin (AF0235, Affinity), which were incubated at 4 °C overnight. The membrane was then treated with a secondary antibody at room temperature for 1 h. The signals were detected by using a chemiluminescence system and captured with an imaging instrument.

### 2.7 Live/Dead staining of BMSCs

Following the respective treatments, BMSCs were carefully rinsed with warm PBS. The cells were then incubated with a working solution containing 2 µM calcein-AM and 4.5 µM propidium iodide (PI) for 15–20 min at 37 °C away from light. After incubation, the cells were gently washed with PBS to remove the excess dye. Fluorescence microscopy was performed immediately to visualize the stained cells, where the live cells fluoresced green due to intracellular esterase activity cleaving Calcein-AM, and the dead cells with compromised membranes fluoresced red due to PI binding to nuclear DNA.

### 2.8 Immunofluorescence (IF) staining

For IF staining of A-BMSCs, the cells were seeded on coverslips in a 24-well plate at a density of 1 × 10^4^ cells/mL. Following adherence and subsequent treatment with Fmito or Fmito@ACs, the cells were fixed with 4% paraformaldehyde for 15 min at room temperature. The cells were permeabilized with 0.5% Triton X-100 for 15 min at room temperature, washed with PBS, and blocked with 5% BSA for 1 h at room temperature. The membrane was first treated with primary antibodies anti-P21 (282248-1-AP, Proteintech) and anti-γH2A.X (10856-1-AP, Proteintech) at 4 °C overnight, followed by PBS washes, and a subsequent treatment with corresponding fluorescent secondary antibodies for 1 h at room temperature in the dark. Finally, the coverslips were washed, mounted with DAPI-containing anti-fade medium, and imaged under a confocal laser scanning microscope.

### 2.9 Fracture model construction

All surgical procedures were performed under sterile conditions as per the institutional guidelines. After anesthetizing 18-month-old C57BL/6 mice *via* intraperitoneal injection of pentobarbital sodium (50 mg/kg), the right hind limb was shaved and disinfected with alternating povidone-iodine and 70% ethanol scrubs. A longitudinal incision was created over the medial aspect of the tibia, followed by blunt dissection through the musculature to expose the tibial shaft without periosteal stripping. A standardized closed fracture was then created at the mid-diaphysis by using the three-point bending technique with custom-made forceps after pre-drilling a pilot hole. The fracture was immediately stabilized by inserting a 0.5-mm stainless steel pin in a retrograde fashion through the proximal tibial plateau into the medullary cavity. Fracture completeness and alignment were then verified through postoperative X-ray imaging. Experimental interventions commenced 3 days after the surgical procedure. All animals were permitted unrestricted cage activity and monitored daily until the scheduled endpoints.

### 2.10 *Ex vivo* and *in vivo* biodistribution analysis

A single 200-μL dose of Fmito@ACs was delivered to mice via a tail vein injection (1.0 × 10^5^ particles/mL). To track their systemic distribution, the animals were anesthetized with 2% isoflurane and physiologically maintained on a heating stage. Whole-body fluorescence imaging was conducted at 24-h post-injection by using a PerkinElmer IVIS Spectrum system, and the quantitative analysis was performed by using Living Image 5.0 software. At this terminal time point, the mice were euthanized *via* cervical dislocation under anesthesia, followed by the collection of major organs (such as the heart, liver, spleen, kidney, and lungs) and bones (i.e., femur and tibia) for *ex vivo* fluorescence imaging under consistent parameters to assess the tissue-specific accumulation.

### 2.11 Bone tissue sectioning and staining

Fresh bone tissue samples were fixed in 4% paraformaldehyde for 24 h. The tissues were then decalcified in 10% EDTA solution (pH 7.4) for 3 weeks at 4 °C; the decalcification solution was changed regularly until no resistance was felt upon needle penetration. The fully decalcified tissue blocks were rinsed under running water and sequentially dehydrated in 15% and 30% sucrose solutions, followed by embedding in an OCT compound and serial sectioning into 8-μm-thick slices using a −20 °C cryostat. The prepared sections were mounted on anti-off slides and stored at −20 °C. For staining, the sections were returned to room temperature and sequentially stained with hematoxylin solution for 5 min, rinsed under running water, differentiated in 1% acid alcohol for a few seconds, and stained in a bluing solution. This was followed by counterstaining with Eosin solution for 3 min. Meanwhile, other sections were sequentially stained with Fast Green solution for 5 min, rinsed briefly in 1% acetic acid for differentiation, and then stained with Safranin O solution for 3 min. Following the staining procedures, the sections were dehydrated through a graded ethanol series, cleared in xylene, and subsequently mounted. The images were finally observed and captured using an optical microscope.

### 2.12 Bulk RNA transcriptomics

Callus tissues harvested at day 14 post-fracture were snap-frozen in liquid nitrogen and stored at −80 °C until processing. Total RNA was isolated with a TRIzol reagent, and its integrity was verified on the Agilent 2,100 Bioanalyzer. Sequencing libraries were constructed following Illumina’s standard protocol. Briefly, poly(A)+ RNA was enriched with oligo (dT) beads, fragmented, and reverse-transcribed into cDNA. The resulting cDNA underwent end repair, adenylation, and adapter ligation, followed by PCR amplification. The final libraries were quality-controlled for size distribution by using the Bioanalyzer before sequencing them on the Illumina NovaSeq 6,000 platform (PE150) to generate high-quality data for downstream analysis.

### 2.13 Statistical analysis

Statistical analyses were conducted using GraphPad Prism 9.0 and ImageJ software, with data presented as the mean ± standard deviation (mean ± SD). Differences between the two groups were assessed by Student’s t-test, whereas one-way analysis of variance (ANOVA) was employed for multi-group comparisons. *P* < 0.05 was considered to indicate statistical significance.

## 3 Result

### 3.1 Preparation and characterization of dual thermo-magnetic responsive Fmito@ACs

**FIGURE 1:**
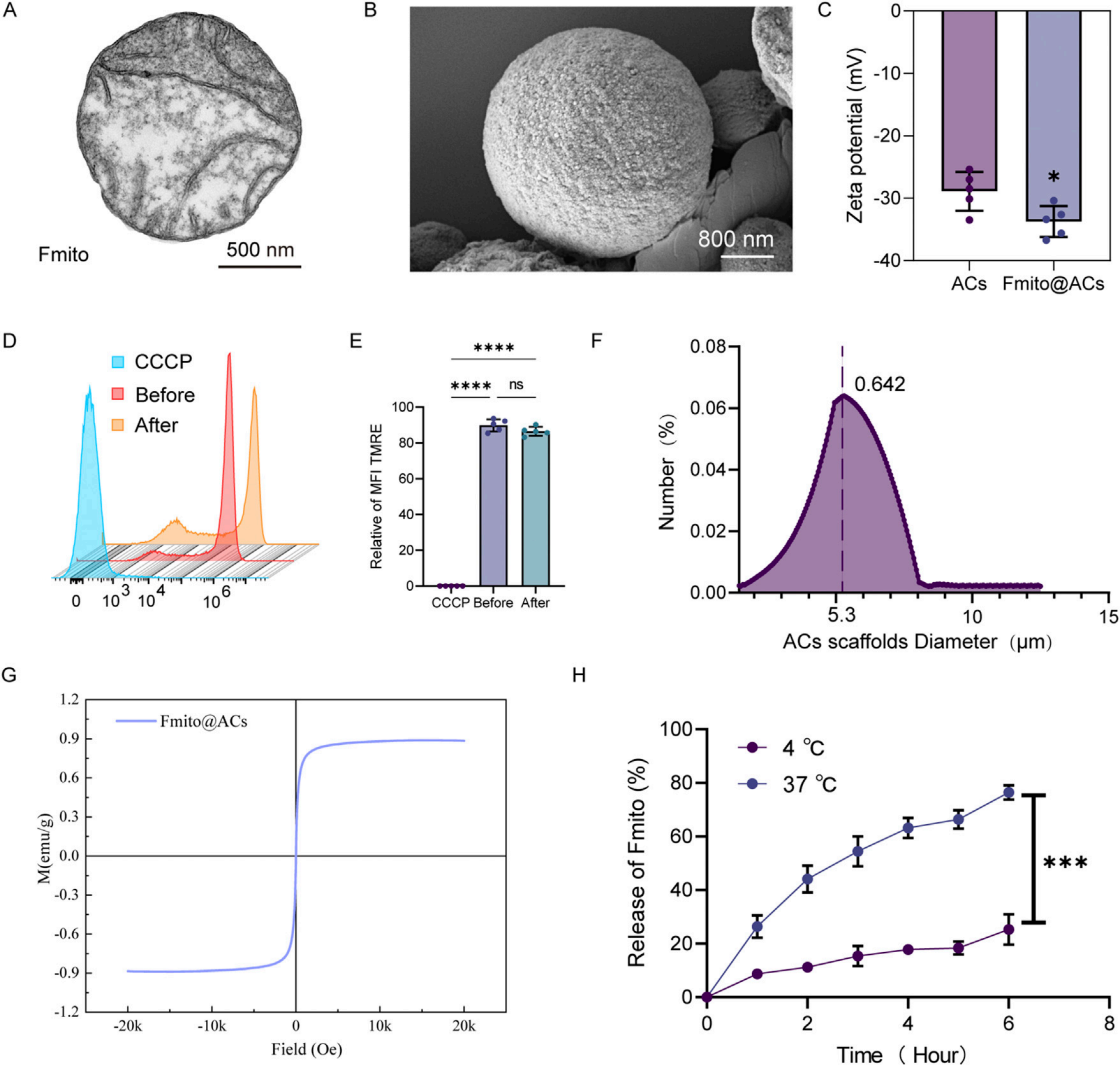
Characterization of artificial cells. **(A)** TEM image showing isolated mitochondria with intact morphology. **(B)** Representative scanning electron microscopy (SEM) image of Fmito@ACs. **(C)** The zeta potential of Fmito@ACs analyzed *via* dynamic light scattering (*n* = 5). **(D)** Representative flow cytometry histograms and **(E)** quantitative analysis of TMRE fluorescence, demonstrating the membrane potential in mitochondria isolated before and after delivery (*n* = 5). **(F)** Hydrodynamic diameter distribution of the prepared ACs in an aqueous solution, as determined by dynamic light scattering. **(G)** Hysteresis loop of the ACs measured by a vibrating sample magnetometer (VSM), demonstrating their magnetic properties. **(H)** *In vitro-*release kinetics profile of Fmito from the ACs under different temperature conditions (4 °C and 37 °C) over time (*n* = 6). All statistical data are presented as the mean ± SD. Statistical analyses were performed using an unpaired, two-tailed Student’s t-test. *P < 0.05, ***P < 0.001, ****P < 0.0001.

Fmito were isolated with a mitochondrial extraction kit and characterized by transmission electron microscopy (TEM). The TEM images revealed structurally intact mitochondria with continuous outer membranes and clearly defined cristae formed by the inner membrane (Figure 1A). We subsequently constructed Fmito@ACs, in which free Fmito were encapsulated within a GelMA hydrogel core to ensure structural protection, followed by surface modification with phosphatidylserine (PS) to introduce an “eat-me” signal that promotes cellular uptake *in vivo* and enhances mitochondrial delivery efficiency (Chu et al., 2025). The Fmito@ACs were solidified through UV cross-linking and subjected to comprehensive characterization (Figure 1B). Zeta potential analysis revealed that Fmito@ACs exhibits a moderately negative charge (−33.74 mV), generating electrostatic repulsion that contributes to its colloidal stability in aqueous media (Figure 1C). To confirm the functional integrity of mitochondria before and after delivery, we performed TMRE staining. The results confirming the preservation of functional integrity throughout the process (Figures 1D,E). Size distribution analysis showed that the resulting microspheres had a uniform diameter of approximately 6.5 μm, comparable to that of normal red blood cells, allowing potential free circulation within the bloodstream (Figure 1F). Further analysis confirmed that the microspheres exhibited strong magnetic responsiveness (Figure 1G). *In vitro* release kinetics further demonstrated a significantly accelerated release of Fmito from ACs at 37 °C, with a cumulative release of 75.36% over 7 days, compared with only 25.66% at 4 °C (Figure 1H). This temperature-responsive release profile enables stable storage of the material *ex vivo* while ensuring efficient mitochondrial release in the physiological *in vivo* environment. In summary, we successfully fabricated an artificial cell platform for mitochondrial delivery featuring both magnetic responsiveness and temperature-triggered release capability under physiological conditions.

### 3.2 Fmito@ACs play a dual role *in vitro* by promoting osteogenesis and inhibiting osteoclastogenesis

**FIGURE 2:**
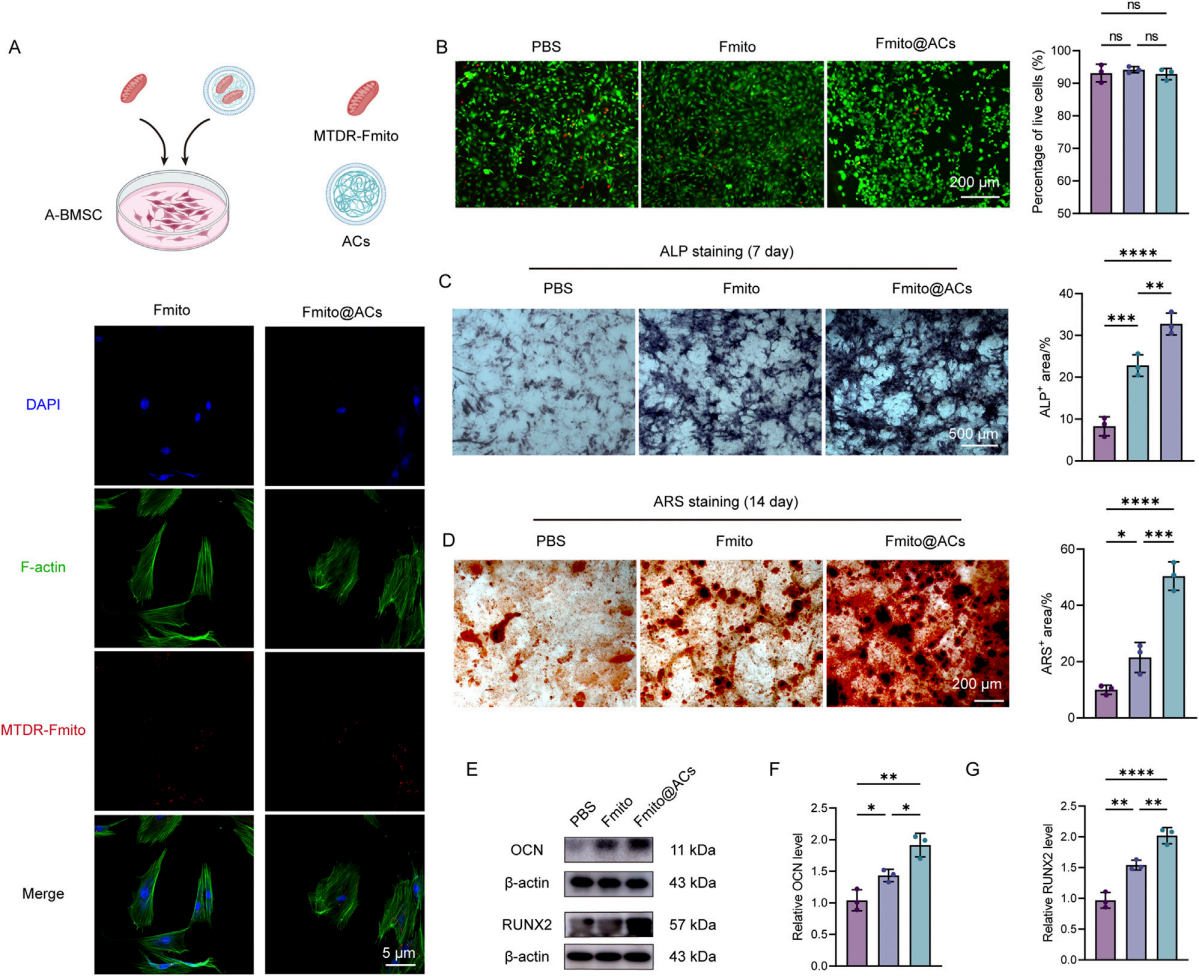
Fmito@ACs promote osteogenic differentiation and inhibit osteoclastogenesis. **(A)** Fluorescent micrographs showing the uptake of Fmito released from ACs by A-BMSCs. Fmito were pre-labeled with MTDR (red). **(B)** Representative Live/Dead staining images of A-BMSCs following treatment with ACs. Live cells stain green (Calcein-AM), and dead cells stain red (propidium iodide) (*n* = 3). **(C)** ALP staining and corresponding quantitative analysis of the cells after 7 days of osteogenic induction (*n* = 3). **(D)** ARS staining and its quantitative results demonstrating mineralized nodule formation after 14 days of osteogenic induction (*n* = 3). **(E–G)** Western blotting displaying the protein expressions of osteogenesis-related markers (OCN and RUNX2), along with statistical analysis of their relative expressions (*n* = 3). All statistical data are presented as the mean ± SD. Statistical analyses were performed using one-way ANOVA with Bonferroni’s *post hoc* test. *P < 0.05, **P < 0.01, ***P < 0.001, ****P < 0.0001.

To examine the interaction between Fmito@ACs and A-BMSCs, Fmito were labeled with MitoTracker Deep Red (MTDR) and co-cultured with A-BMSCs. Confocal microscopy after 24 h of incubation showed that 86.65% of A-BMSCs internalized free Fmito, while 91.33% successfully internalized Fmito@ACs, indicating that ACs encapsulation did not impair the cellular uptake efficiency of Fmito and supporting their functional potential *in vivo* (Figure 2A; Supplementary Figure S1B). To assess whether functional Fmito could be internalized and sustained within A-BMSCs, MTR-labeled Fmito loaded in ACs were co-cultured with the cells. Results showed that active Fmito persisted for over 4 h post-delivery (Supplementary Figure S1A). Live/Dead staining confirmed that neither empty ACs nor Fmito@ACs exhibited significant cytotoxicity toward A-BMSCs compared with the PBS control (Figure 2B). Considering that fracture healing requires coordinated new bone formation and osteoclast-mediated bone remodeling (González Díaz et al., 2023), we next assessed the effects of Fmito and Fmito@ACs on osteogenic and osteoclast differentiation *in vitro*. Both treatments significantly enhanced osteogenic differentiation at days 7 and 14 compared with PBS, with Fmito@ACs showing the greatest pro-osteogenic activity (Figures 2C,D). The upregulation of osteogenesis-related proteins OCN and RUNX2 further confirmed these findings (Figures 2E–G). Moreover, Fmito@ACs significantly inhibited osteoclast differentiation, which may promote bone formation during the early phase of fracture healing (Supplementary Figures S1C–E). Collectively, these results demonstrate that Fmito@ACs are efficiently internalized by A-BMSCs with excellent biocompatibility *in vitro* and exhibit dual functionality by promoting osteogenic differentiation while suppressing osteoclastogenesis.

### 3.3 Fmito@ACs alleviated A-BMSCs senescence by enhancing mitochondrial oxidative phosphorylation

**FIGURE 3:**
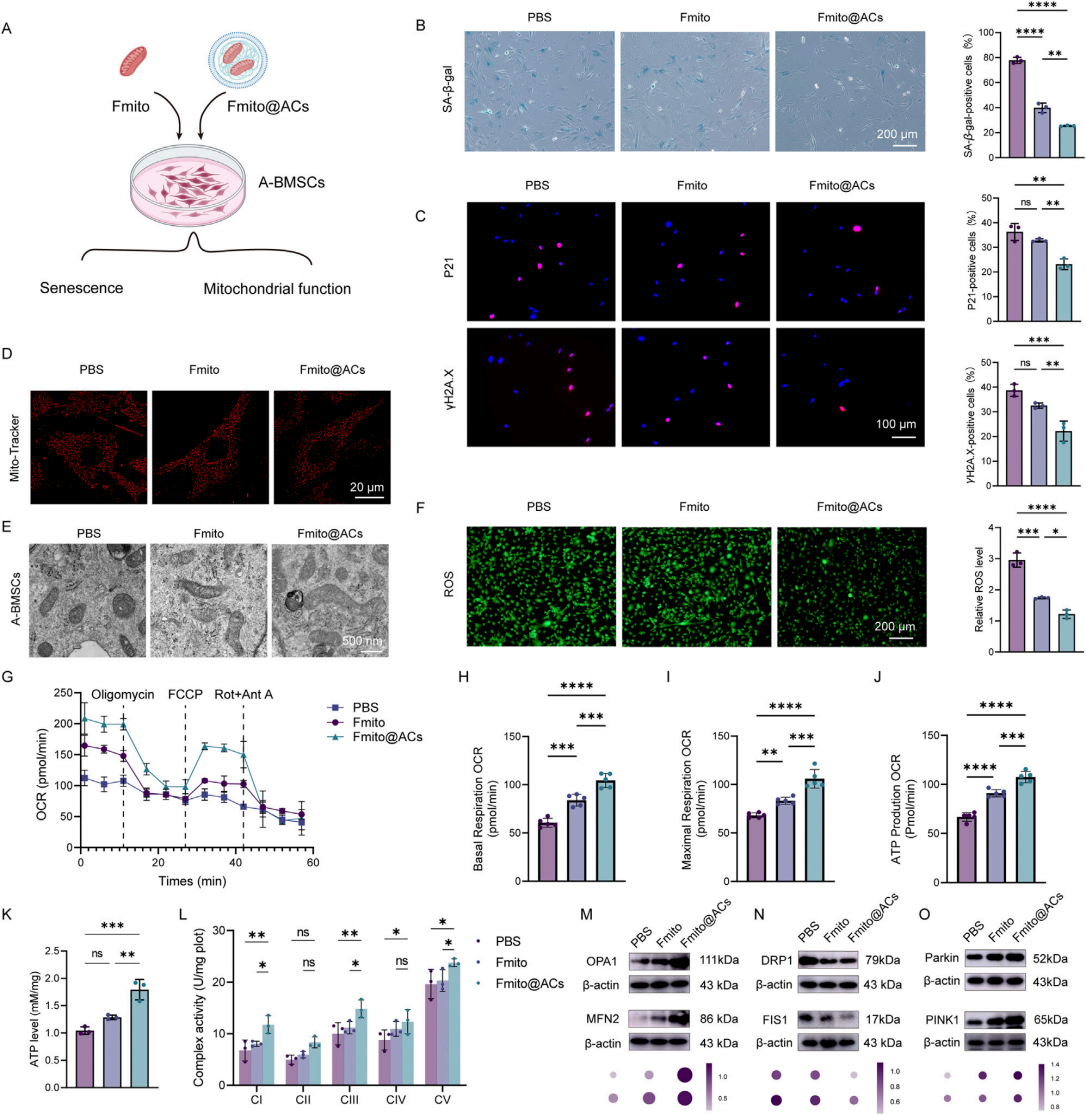
Fmito@ACs reverse the senescent phenotype and restore mitochondrial function in A-BMSCs. **(A)** Schematic illustration of the experimental design for treating A-BMSCs with Fmito@ACs. **(B)** Representative images of SA-β-gal staining and the corresponding quantitative analysis of A-BMSCs (*n* = 3). **(C)** Immunofluorescence staining images and quantitative analysis of senescence-associated markers (P21 and γH2A.X) in A-BMSCs (*n* = 3). **(D)** Representative fluorescent images of the mitochondria in A-BMSCs stained with MTDR. **(E)** TEM images displaying the mitochondrial ultrastructure in A-BMSCs. **(F)** Representative images of intracellular ROS detection and statistical analysis of the fluorescence intensity (*n* = 3). **(G)** Measurement of OCR in A-BMSCs by using the Seahorse XF Mitochondrial Stress Assay Kit (*n* = 5). **(H–J)** Statistical analysis of basal respiration, maximal respiration, and ATP production capacity derived from OCR measurements in different groups (*n* = 5). **(K)** Measurement of ATP levels in A-BMSCs (*n* = 3). **(L)** Activities of CI-CV from A-BMSC (*n* = 3). **(M–O)** Representative Western blot images of key proteins in mitochondrial dynamics, including fusion (OPA1, MFN2), fission (DRP1, FIS1), and mitophagy (PINK1, Parkin) (*n* = 3). All statistical data are presented as the mean ± SD. Statistical analyses were performed using one-way ANOVA with Bonferroni’s *post hoc* test or unpaired, two-tailed Student’s t-test. *P < 0.05, **P < 0.01, ***P < 0.001, ****P < 0.0001.

Mitochondrial dysfunction in A-BMSCs critically impairs their osteogenic differentiation potential. To evaluate the restorative effects of Fmito@ACs on A-BMSCs, we conducted a series of *in vitro* assays (Figure 3A). Senescence-associated β-galactosidase (SA-β-Gal) staining showed that Fmito@ACs treatment significantly reduced the proportion of SA-β-Gal-positive A-BMSCs (Figure 3B). Consistently, the expression of senescence markers P21 and γH2A.X was significantly downregulated (Figure 3C). To further assess mitochondrial changes, we examined morphology and function using TEM and MitoTracker fluorescence staining. Compared with the PBS and free Fmito groups, Fmito@ACs treatment effectively restored normal mitochondrial morphology in A-BMSCs. Mitochondria in the Fmito@ACs group displayed continuous outer membranes and clearly defined cristae, whereas those in the PBS and free Fmito groups exhibited significant mitochondrial swelling and disorganized, blurred cristae (Figures 3D,E). Functionally, Fmito@ACs treatment significantly reduced intracellular reactive oxygen species (ROS) levels, showing stronger antioxidative effects than both control groups (Figure 3F).

Following a fracture, the local hypoxic microenvironment further exacerbates mitochondrial function, impeding healing. Using the Seahorse XF Analyzer, we measured oxygen consumption rate (OCR) in cells from different groups. The results indicated that Fmito@ACs treatment significantly improved basal and maximal respiration, ATP production, and spare respiratory capacity in A-BMSCs, outperforming free Fmito across all parameters (Figures 3G–J). This enhancement of aerobic respiratory capacity supports BMSC differentiation and osteogenesis (Luo et al., 2025). ATP bioluminescence detection confirmed significantly increased ATP levels in A-BMSCs treated with Fmito@ACs (Figure 3K). The levels of several respiratory enzymes were also significantly increased, especially respiratory complex I and III (Figure 3L). Mitochondrial dynamics is an important measure of mitochondrial function (Chen et al., 2025). We examined the expression of proteins involved in mitochondrial fusion (MFN2 and OPA1) and fission (DRP1 and FIS1) (Figures 3M,N). The results demonstrated an upregulation of pro-fusion proteins and a downregulation of pro-fission proteins in the mitochondrial transplantation group. Besides, the PINK1/Parkin mitophagy pathway was markedly activated (Figure 3O). Collectively, these findings indicate that Fmito@ACs restore mitochondrial homeostasis by promoting fusion and mitophagy, effectively reversing the senescent phenotype of A-BMSCs and enhancing their osteogenic potential.

### 3.4 Fmito@ACs exhibit favorable tissue targeting and biosafety

**FIGURE 4:**
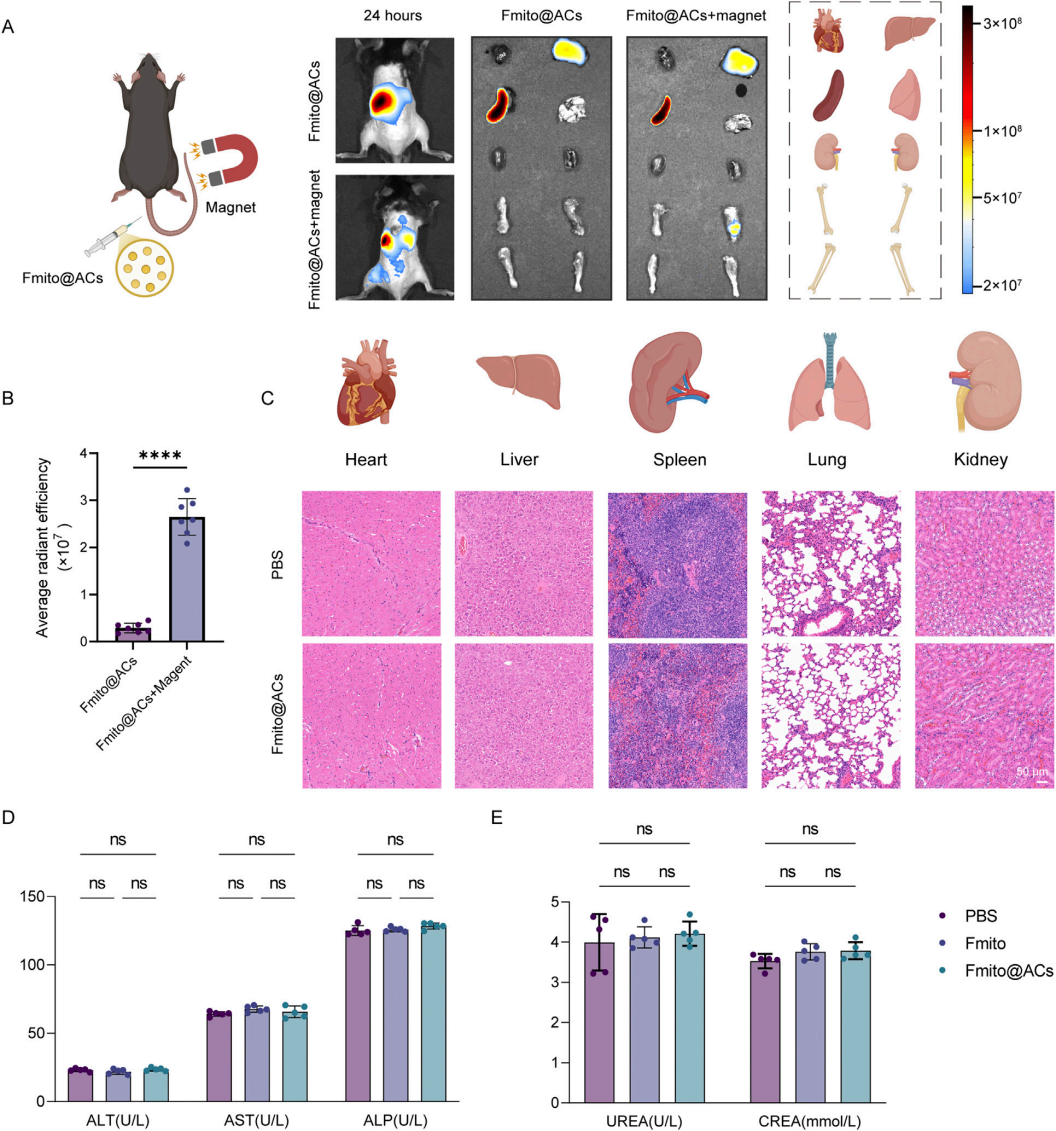
Fmito@ACs possess magnetic targeting function and biological safety *in vivo.* **(A,B)** Representative IVIS imaging results following Fmito@ACs injection, displaying biodistribution and fracture site accumulation (*n* = 7). **(C)** H&E staining of the heart, liver, spleen, lung, and kidney tissues post-administration of Fmito@ACs (*n* = 5). **(D,E)** Bar graph showing the comparison of hepatic and renal function parameters between mice receiving intravenous Fmito@ACs and the control group (*n* = 5). Statistical analyses were performed using one-way ANOVA with Bonferroni’s *post hoc* test or unpaired, two tailed Student’s t-test. ****P < 0.0001.

We next assessed the *in vivo* therapeutic efficacy of Fmito@ACs administered through tail vein injection. At 24 h postinjection, IVIS imaging under an external magnetic field applied to the fracture site revealed prominent accumulation of Fmito@ACs at the fracture region, along with moderate distribution in metabolic organs such as the liver and spleen, confirming successful magnetically guided targeting (Figures 4A,B). To evaluate the biosafety of Fmito@ACs, mice were administered consecutive injections for 3 weeks. Subsequent analysis revealed no significant differences in hepatic and renal function parameters or in the histopathological morphology of major organs (including the heart, liver, spleen, lungs, and kidneys) between the treatment and control groups. These results collectively indicate that Fmito@ACs exhibit a favorable biosafety profile (Figures 4C–E).

### 3.5 Fmito@ACs promote the healing ofage-related fractures *in vivo*

**FIGURE 5:**
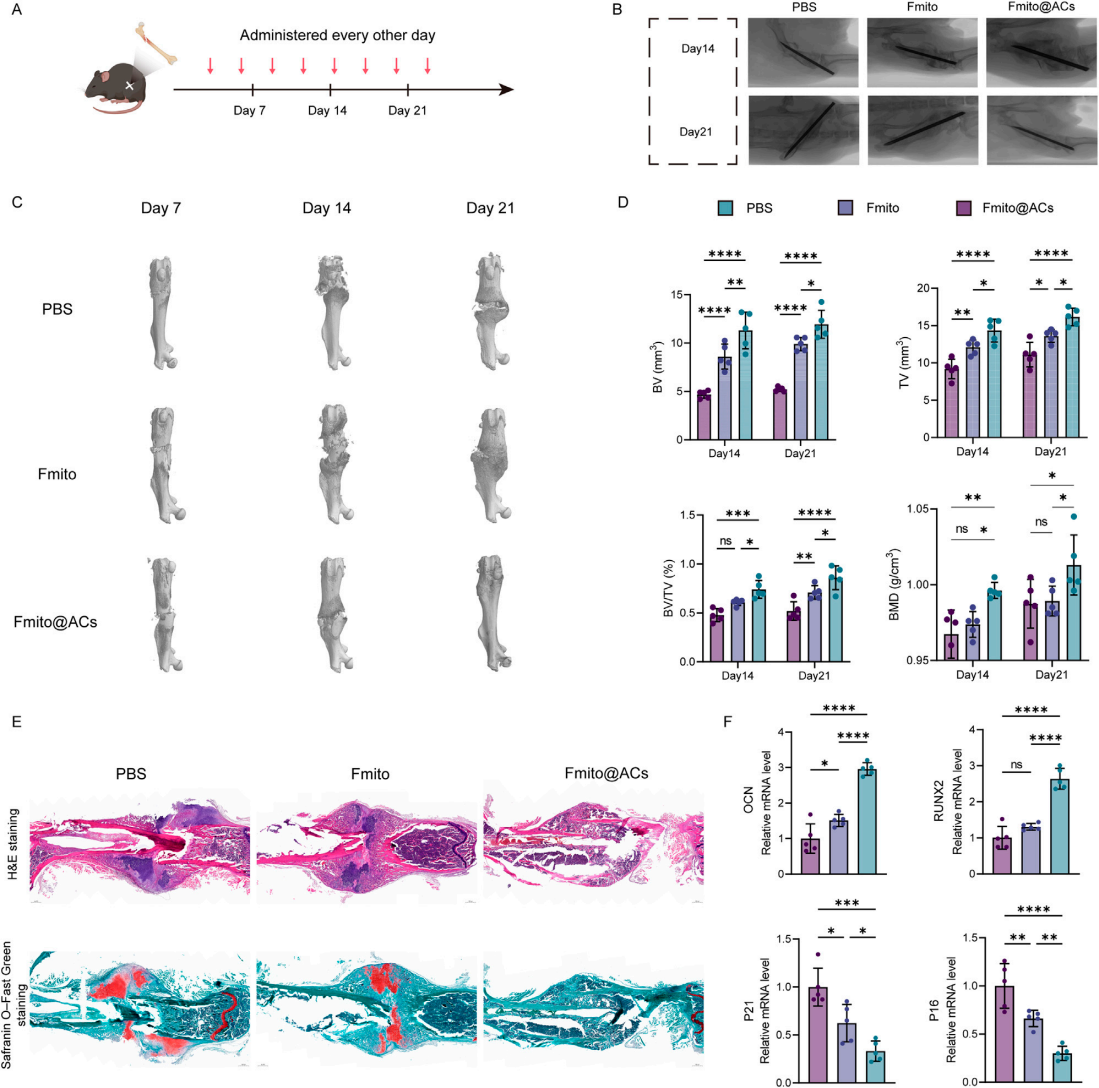
Systemic administration of Fmito@ACs promotes the healing of age-related fractures *in vivo*. **(A)** Schematic illustration of the *in vivo* Fmito@ACs injection protocol. **(B)** X-ray images of the fracture sites at days 14 and 21 post-injections. **(C)** Representative micro-CT reconstruction images of the fracture regions at days 7, 14, and 21 post-injections. **(D)** Quantitative micro-CT analysis of the bone-regeneration parameters at days 14 and 21 (*n* = 5). **(E)** H&E and Safranin O/Fast Green staining of the fracture areas at day 21, exhibiting enhanced bone formation and matrix organization (*n* = 5). **(F)** qPCR analysis of osteogenesis-related and senescence-associated gene expression in the callus tissues (*n* = 5). All statistical data are presented as the mean ± SD. Statistical analyses were performed using one-way ANOVA with Bonferroni’s *post hoc* test or unpaired, two-tailed Student’s t-test. *P < 0.05, **P < 0.01, ***P < 0.001, ****P < 0.0001.

To further assess the reparative effects of Fmito@ACs on age-related fractures, mice received Fmito@ACs *via* tail vein injection every other day, followed by a 30-min external magnetic field application after each injection (Figure 5A). Fracture healing was evaluated on days 7, 14, and 21. Micro-computed tomography (micro-CT) and X-ray imaging revealed significantly enhanced callus formation and nearly complete disappearance of fracture lines in the Fmito@ACs group, whereas residual fracture gaps were still evident in the PBS and free Fmito groups, indicating a strong pro-healing effect of Fmito@ACs (Figures 5B–D). Histological analysis corroborated these findings: H&E staining demonstrated denser, more continuous bone structures in the Fmito@ACs group at day 21, while Safranin O/Fast Green staining showed greater ossification (Figure 5E). At the molecular level, gene expression analysis of bone tissue revealed that Fmito@ACs significantly upregulated osteogenesis-related genes and concurrently downregulated senescence-associated genes (Figure 5F). Senescent skeletal stem cells exacerbate the inflammatory milieu of the bone marrow by secreting senescence-associated secretory phenotype (SASP) factors, which impede fracture healing (Ambrosi et al., 2021). Consistent with this, quantification of inflammatory cytokines (interleukin-1 beta, interleukin-6, interleukin-8, and tumor necrosis factor-alpha) showed that Fmito@ACs treatment significantly reduced the secretion of SASP-related inflammatory mediators (Supplementary Figure S2A). Collectively, these results demonstrate that Fmito@ACs significantly accelerate fracture healing and induce rejuvenation in age-related fractures *in vivo*.

### 3.6 Fmito@ACs enhance fracture healing capacity in aging

**FIGURE 6:**
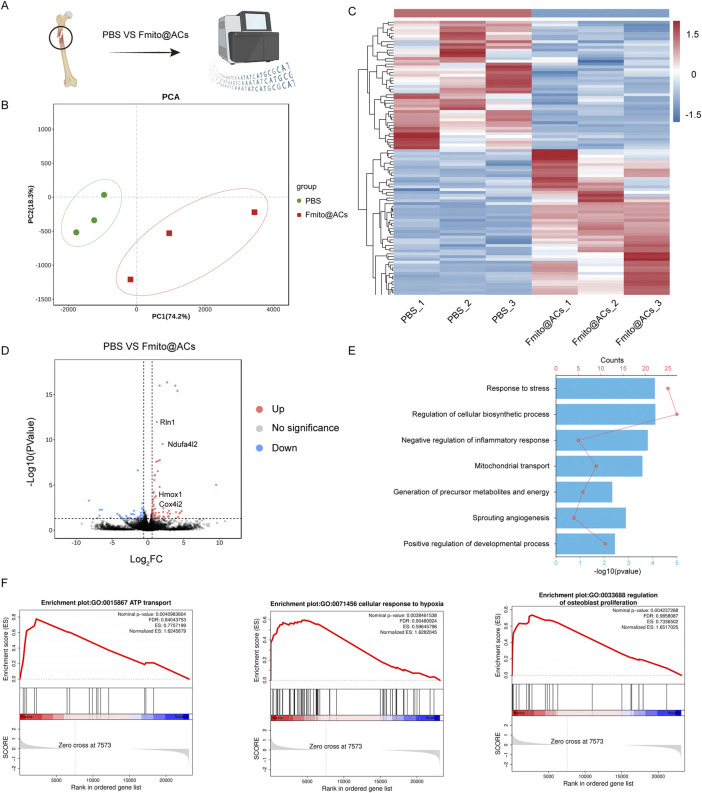
Fmito@ACs administration enhances osteogenesis and improves energy metabolism *in vivo.* **(A)** A schematic workflow of bulk RNA-sequencing analysis performed on fracture callus tissues. **(B)** PCA plot exhibiting the transcriptomic profiles of different treatment groups. **(C)** Heatmap visualization of DEGs between the experimental groups. **(D)** Volcano plot displaying significantly upregulated (red) and downregulated (blue) genes. **(E)** GO enrichment analysis of biological processes significantly altered by Fmito@ACs treatment. **(F)** GSEA plots demonstrating enrichment of the key metabolic and osteogenic pathways.

To elucidate the molecular mechanisms underlying the effects of Fmito@ACs on age-related fracture healing, transcriptome sequencing was performed on callus tissues collected 14 days postfracture (Figure 6A). Principal component analysis (PCA) showed a distinct separation between the PBS and Fmito@ACs groups at the transcriptome level, demonstrating extensive gene expression reprogramming induced by the treatment (Figure 6B). Furthermore, heatmap analysis confirmed widespread transcriptional differences between the two groups (Figure 6C). Differential expression analysis identified 1,468 differentially expressed genes (DEGs) (Log2 |FC| > 1, *P* < 0.05), encompassing multiple functional categories, including fracture healing–related genes (e.g., *RLN*, *VEGFA*, *MMP9*), genes involved in energy metabolism (e.g., *Ndufa4l2*, *Cox4i2*, *PGK1*), and genes mediating adaptation to hypoxic stress (e.g., *Hmox1*, *EGR1*, *NDRG1*) (Figure 6D). Gene Ontology (GO) enrichment analysis showed that these DEGs were significantly enriched in biological processes associated with stress response, inflammatory signaling inhibition, mitochondrial transport, and osteogenic differentiation (Figure 6E). Moreover, gene set enrichment analysis (GSEA) demonstrated pronounced activation of ATP transport (GO:0015867), cellular response to hypoxia (GO:0071456), and osteogenesis-associated processes (GO:0033688) in the Fmito@ACs group (Figure 6F). Collectively, these findings indicate that Fmito@ACs enhance fracture repair in aged mice through a coordinated regulatory mechanism that augments local energy metabolism, improves hypoxia adaptation, mitigates inflammation in the bone marrow niche, and ultimately promotes osteogenesis.

## 4 Discussion

We herein successfully developed Fmito@ACs, incorporating Fmito, and demonstrated its efficacy in promoting age-related fracture healing by restoring mitochondrial function in aged tissue. The findings provide not only an innovative strategy for treating age-related bone repair disorders but also new insights into the intervention mechanism of bone aging from the viewpoint of cellular bioenergetics.

Mitochondria are central to cellular energy metabolism, and their functional decline is recognized as a hallmark of aging (Shu et al., 2025). Such dysfunction, characterized by increased ROS levels, decreased mitochondrial membrane potential, and impaired ATP production, interrupts mitochondrial homeostasis and contributes to bone pathology (de la Mata et al., 2016). Our findings demonstrate that aged mitochondria exhibit pronounced structural abnormalities and functional decline, leading to bioenergetic insufficiency that directly compromises osteogenic differentiation. This underscores mitochondrial dysfunction as a key driver of delayed fracture healing and supports the therapeutic potential of restoring mitochondrial activity. Intercellular mitochondrial transfer, a naturally occurring process, has been reported in both physiological and pathological contexts: prostate cancer cells acquire mitochondria from neurons to support metabolism (Hoover et al., 2025), whereas astrocytes transfer mitochondria to neurons to reduce ischemic damage (Falchi et al., 2013). These findings have inspired the concept of mitochondrial supplementation, which enhances immune modulation in T cells and proliferation in vascular endothelial cells. In the skeletal system, mitochondrial transplantation improves aerobic metabolism and osteogenic differentiation in BMSCs (Guo et al., 2020). However, its application in A-BMSCs remains insufficiently studied. Our results show that Fmito transplantation alleviates senescence-associated phenotypes in A-BMSCs, demonstrating the feasibility of using young donor mitochondria to rejuvenate aged cells.

Despite its promise, mitochondrial transfer *in vivo* remains challenging. Free mitochondria are easily damaged by harmful components in the blood and extracellular fluid, such as ROS and high calcium, leading to dysfunction and structural breakdown (Stier, 2021; Westensee et al., 2021). Therefore, an effective scaffold is essential to preserve mitochondrial function. Hydrogels can protect *in vivo* mitochondrial activity and are biocompatible. Gelatin-based hydrogels maintain mitochondria in a state similar to intracellular conditions, allowing sustained ATP production for over 24 h (Westensee et al., 2021). On this basis, gelatin-based hydrogel microspheres were used in this study as protective carriers for mitochondrial delivery.

Fracture repair often requires the participation of stem cells, whose functions are tightly regulated by the surrounding microenvironment (Pandey et al., 2022). After fracture, vascular injury, and hematoma formation reduce local oxygen supply, resulting in impaired osteogenic activity (Lv et al., 2023). Among them, the energy deficiency caused by hypoxia further diminishes osteogenic ability. Fmito implantation may indirectly activate the endogenous repair mechanism by enhancing the tolerance and adaptability of aged cells to a harsh microenvironment. Moreover, senescent cells promote surrounding tissue degeneration by secreting SASP (Demaria et al., 2015), creating a locally degenerated inflammatory microenvironment. Fracture healing is a complex process that depends on interactions within the surrounding environment and is closely influenced by adjacent tissues such as bone marrow and muscle (Zhou et al., 2021). SASP secreted by bone marrow senescent stem cells impairs osteogenesis and promotes osteoclasts, underscoring the importance of targeting aging-related inflammation. Fmito transplantation can significantly inhibit the formation of an inflammatory microenvironment, although the precise underlying mechanism needs to be further explored.

However, it is important to acknowledge several limitations of this study. Firstly, it should be noted that this study is limited to preclinical validation in rodent models. Further investigation in large animal models is necessary to strengthen the translational potential of our findings. Secondly, biomechanical testing, a critical indicator of bone healing quality, was not performed and warrants further investigation. Finally, while Fmito@ACs promoted fracture repair in this model by rejuvenating A-BMSCs, their therapeutic potential in other bone disease models remains to be explored, and this represents a primary direction for our future research.

In conclusion, this study demonstrates that Fmito transplantation improves aging-related phenotypes of A-BMSCs and enhances their adaptability to the hypoxic microenvironment after fracture. The hydrogel microsphere-based delivery system provides an effective strategy to overcome mitochondrial instability and targeting problems during mitochondrial transfer, providing an experimental basis for the treatment of age-related fractures.

## Data Availability

The raw sequence data reported in this paper have been deposited in the Genome Sequence Archive (Genomics, Proteomics & Bioinformatics 2025) in National Genomics Data Center (Nucleic Acids Res 2025), China National Center for Bioinformation / Beijing Institute of Genomics, Chinese Academy of Sciences (GSA: CRA034262) that are publicly accessible at https://ngdc.cncb.ac.cn/gsa/search?searchTerm=CRA034262.
