# Supplementary material for: Bone-targeted mitochondrial delivery via magnetic-temperature responsive artificial cells for repairing age-related fractures
Source: Front Pharmacol. 2026 Jan 2;16:1725973. doi: 10.3389/fphar.2025.1725973 (PMC12808401; doi:10.3389/fphar.2025.1725973)
Supplement: Supplementary file 1 [file DataSheet1.pdf]

Supplementary Information

Supplementary Methods

1.1 Magnetic Targeting Experiment

A solid cylindrical neodymium magnet (N42, with a surface magnetic flux density of ~1.4 T; dimensions: 50 mm × 30 mm × 10 mm) was positioned externally adjacent to the fracture site in the mouse hind limb. The magnetic flux density at the magnet surface was verified using a handheld gaussmeter, measuring 128 mT. Accounting for attenuation through biological tissue, the effective magnetic field strength reaching the fracture site is 113.3 mT after penetrating 1 mm of murine leg tissue, based on an exponential decay model for electromagnetic waves in biological media. The magnetic field gradient was calculated as 15.4 T/m. The magnetic field was applied immediately following the injection of mito@ACs and maintained for 30 minutes.

1.2 Micro-CT Analysis of Bone Tissue

Following a 48-hour fixation in 4% paraformaldehyde, femoral samples were subjected to Micro-CT analysis. All scans were performed at an isotropic voxel size of 8.0 μm using a Skyscan 1276 system (Bruker) to precisely capture the microarchitecture of the callus. The acquired projections were reconstructed into three-dimensional images using NRecon software (v.1.7.3.1). For quantitative assessment of fracture healing, the callus region was defined as the volume of interest (VOI) and analyzed with CTAn software (v.1.18) to determine the following parameters: bone volume (BV), total volume (TV), bone volume fraction (BV/TV), and bone mineral density (BMD).

1.3 Quantitative PCR (qPCR)

Total RNA was extracted from callus tissue using TRIzol reagent, and its concentration and purity were verified with a NanoDrop system. cDNA was synthesized from 1 μg of total RNA using the PrimeScript RT reagent kit. qPCR was performed with SYBR Green Premix Pro Taq HS on a CFX96 Real-Time PCR Detection System, using the following thermal profile: 95°C for 30 s, followed by 40 cycles of 95°C for 5 s and 60°C for 30 s. To avoid potential confounding effects from glucose metabolism pathways, β-Actin was selected as the reference gene, and its stable expression across experimental groups was confirmed using the geNorm algorithm. The relative expression of target genes was calculated using the $2^{-\Delta\Delta Ct}$ method. All primer sequences used are listed in Table S1.

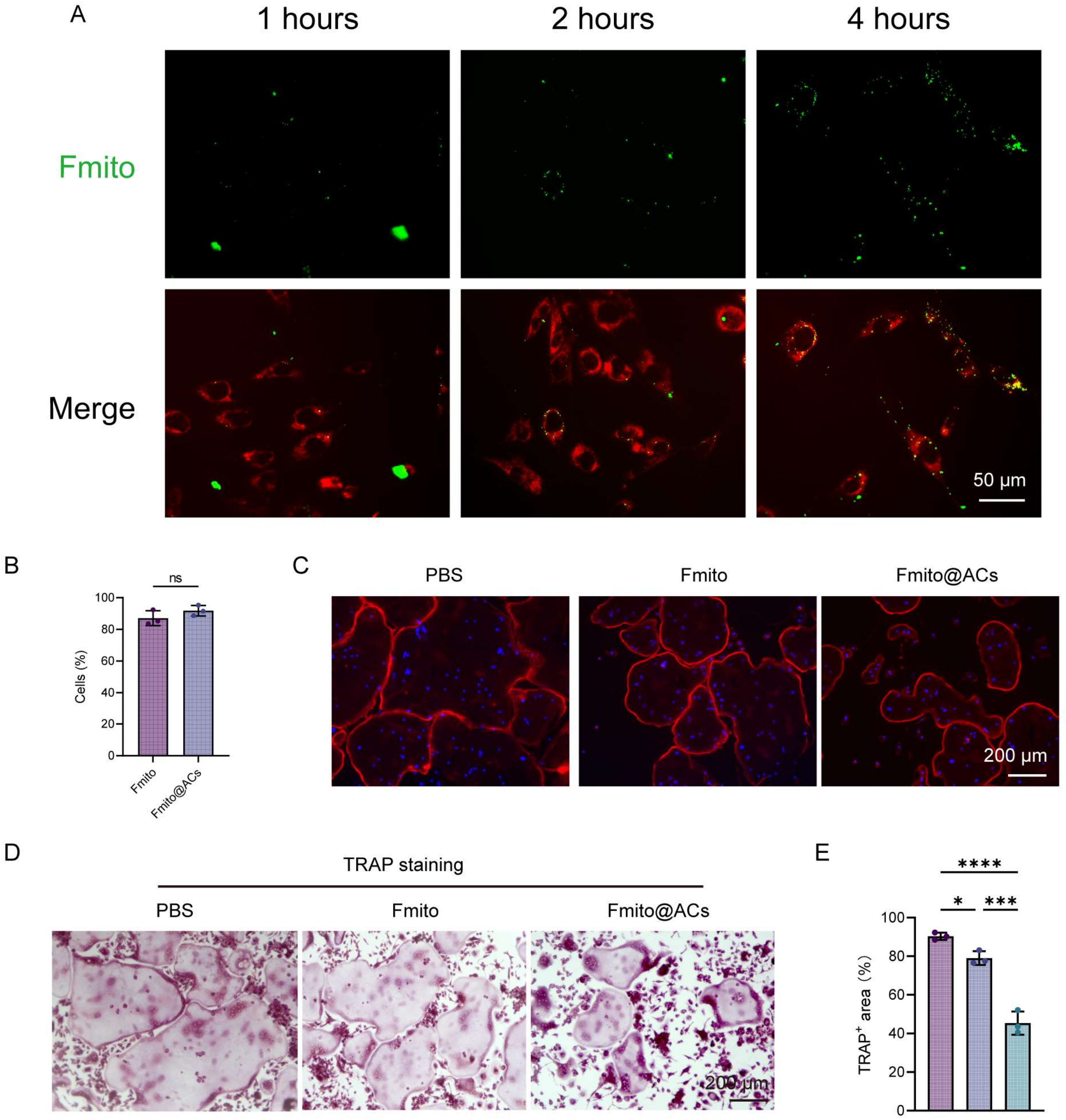

Supplementary Figure 1. Fmito@ACs inhibits osteoclast differentiation. (A) Representative fluorescence microscopy images of the dynamic internalization process of Fmito. Green: MTG labeled Fmito; Red: MTDR labeled endogenous mitochondria. (B) Statistical analysis of the proportion of A-BMSCs internalizing Fmito. (C) Representative fluorescent images of osteoclast actin rings (highlighting multinucleated cells and F-actin structures). (D-E) TRAP staining images and statistical graph of osteoclast differentiation. Statistical analyses were performed using one-way ANOVA with Bonferroni's post-hoc test. *$P < 0.05$, ***$P < 0.001$, ****$P < 0.0001$.

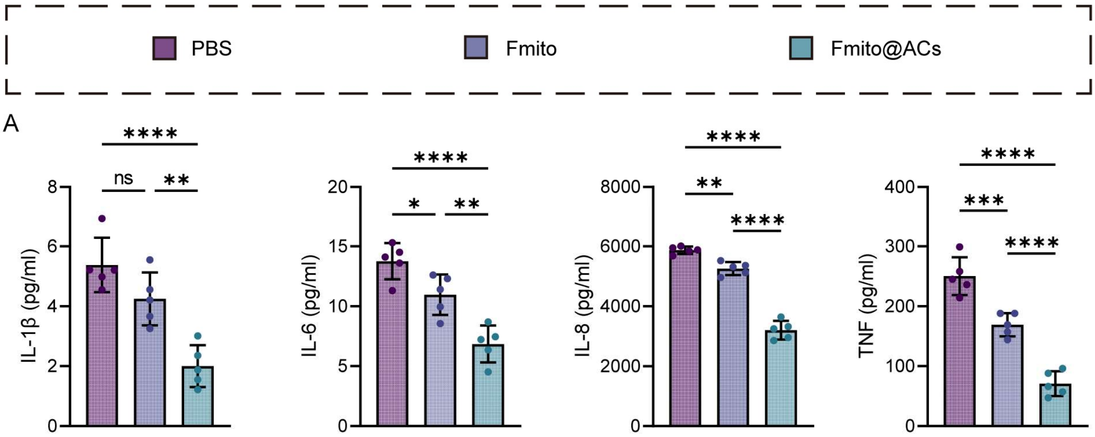

Supplementary Figure 2. Fmito@ACs ameliorate the senescence-associated inflammatory microenvironment. (A) Levels of senescence-associated secretory phenotype (SASP) factors (IL-1β, IL-6, IL-8, TNF-α) in the bone marrow were measured by enzyme-linked immunosorbent assay (ELISA). Statistical analyses were performed using one-way ANOVA with Bonferroni's post-hoc test. $^{*}P < 0.05$, $^{**}P < 0.01$, $^{***}P < 0.001$, $^{****}P < 0.0001$.

Table S1

Gene	Forward primer	Reverse primer
OCN	GGCCCTGAGTCTGACAAAGC	GCTCGTCACAAGCAGGGTTAA
RUNX2	TACCAGCCACCGAGACCAA	AGAGGCTGTTTGACGCCATAG
P21	CAGAATAAAAGGTGCCACAGGC	CGTCTCCGTGACGAAGTCAA
P16	CGTACCCCGATTCAGGTGATG	ACGATGTCTTGATGTCCCCG
